# Selective BuChE Inhibitory Activity, Chemical Composition, and Enantiomeric Content of the Essential Oil from *Salvia leucantha* Cav. Collected in Ecuador

**DOI:** 10.3390/plants10061169

**Published:** 2021-06-09

**Authors:** Gabriela Villalta, Melissa Salinas, James Calva, Nicole Bec, Christian Larroque, Giovanni Vidari, Chabaco Armijos

**Affiliations:** 1 Departamento de Química, Universidad Técnica Particular de Loja, 1101608 Loja, Ecuador; gcvillalta1@utpl.edu.ec (G.V.); masalinas4@utpl.edu.ec (M.S.); jwcalva@utpl.edu.ec (J.C.); 2 Institute for Regenerative Medicine and Biotherapy (IRMB), Université de Montpellier, INSERM, CHU Montpellier, 34295 Montpellier, France; nicole.bec@inserm.fr (N.B.); cjlarroque@gmail.com (C.L.); 3 Medical Analysis Department, Faculty of Science, Tishk International University, Erbil 44001, Iraq; vidari@unipv.it

**Keywords:** *Salvia leucantha*, essential oil, enantiomer distribution, GC-MS, GC-FID, BuChE inhibitory activity

## Abstract

The essential oil (EO) of *Salvia leucantha* Cav. was isolated by steam distillation of the aerial parts collected in the South of Ecuador. Its physical properties were evaluated and the chemical composition of the oil was determined by GC-MS and GC-FID analyses using two chromatographic columns, DB-5ms and HP-INNOWax. Six major compounds were identified, namely, the sesquiterpenes 6.9-guaiadiene (19.14%), *(E)*-caryophyllene (16.80%), germacrene D (10.22%), *(E)*-β-farnesene (10.00%), and bicyclogermacrene (7.52%), and the monoterpenoid bornyl acetate (14.74%). Furthermore, four pairs of enantiomers were determined by enantioselective GC-MS of the essential oil. (−)-germacrene D and (+)-α-pinene showed the highest enantiomeric excess (*ee%*). In an in vitro assay, the essential oil demonstrated an interesting inhibitory activity of the enzyme butyrylcholinesterase (BuChE), with an IC_50_ = 32.60 µg/mL, which is the highest determined for a *Salvia* species. In contrast, the oil was weakly active against acetylcholinesterase (AChE) with an IC_50_ > 250 µg/mL.

## 1. Introduction

The family Lamiaceae comprises about 235 genera and approximately 7170 identified species [1]. *Salvia*, that is the largest genus in the family, is estimated to include about 900 species distributed worldwide [2]. In Ecuador, the family Lamiaceae comprises about 27 genera and 219 species that grow especially in Andean forests, badlands, and dry inter-Andean valleys [3]. Several Lamiaceae have a high pharmacological value, for the production of a wide range of secondary metabolites with antibacterial, antioxidant, anti-inflammatory, antimicrobial, antiviral, and anticancer properties [4].

*Salvia leucantha* Cav. (Mexican bush sage, velvet sage) is a plant native to Mexico that is now distributed worldwide, including across the entire American continent [5]. It is an herbaceous shrub that can reach up to 1.20 m in height; the lanceolate leaf blades are 10–20 cm long and are grayish-green with a velvety texture. It has a purple chalice and purple or white corolla that are covered by thin, velvety hairs. The plant is used in traditional medicine as a remedy to relieve cough, chest, lung, and stomach pains, as well as a garden plant [6,7].

The plant has a pleasant smell due to the presence of a fragrant volatile fraction. According to a few studies carried out in Asia and South America on the chemical composition of the essential oil (EO) from *S. leucantha*, sesquiterpene hydrocarbons have been identified as the predominant components [8]. Moreover, in vitro studies of the EO have shown various properties such as antioxidant, antimicrobial, antispasmodic, as well as digestive and neuroprotective effects [9].

On a general note, thanks to their odorant and biological properties, such as antimicrobial, antioxidant, anti-inflammatory, antiviral, antimutagenic, and anticancer ones [10,11], several EOs have applications not only in fragrance industries and food flavorings, but also in medical and clinical microbiology, pharmaceutical botany, food preservation, and others [12,13]. In this context, considering the various pharmaceutical properties of the components, it is important to also test EO’s activity on less traditional pharmacological targets or biological systems, such as the cholinesterase (ChE) enzymes, acetylcholinesterase (AChE) and butyrylcholinesterase (BuChE). In fact, these enzymes are actively involved in studies aimed at the treatment of neurodegenerative diseases such as Alzheimer’s (AD) [14], neuromuscular disorders such as Myasthenia gravis (MG), and eye pathologies such as glaucoma. Patients treated with ChE inhibitors seem to experience improvements in their cognitive functions; however, the features of the two ChE inhibitors differ considerably [15].

We were interested in comparing the EO isolated from aerial parts of *S. leucantha* collected in Ecuador with the essential oils from samples of the plant collected in other countries. In fact, different chemical compositions may indicate the existence of genotypes. Equally important to us was determining, for the first time, the enantiomeric composition and the in vitro AChE and BuChE inhibitory activities of the EO.

## 2. Results and Discussion

### 2.1. Physical Properties of the EO

The steam-distilled EO from aerial parts of *S. leucantha* presented a pale-yellow color and a pleasant smell. The average yield of three distillations was 0.03 ± 0.01%. The mean relative density and refractive index of the oil were 0.94 ± 0.01 g/mL and 1.49 ± 0.01, respectively.

### 2.2. Chemical Composition of the EO

Forty-two compounds were identified in the EO by using GC-MS and GC-FID analyses. When using a non-polar DB-5ms column, 99.13% of the components present in the EO were identified, while 98.05% of the EO components were identified when using a polar HP-INNOWax column. The analysis on DB-5ms showed that *S. leucantha* EO consists mostly of sesquiterpene hydrocarbons (71.49%), oxygenated monoterpenes (15.12%), monoterpene hydrocarbons (9.48%), oxygenated sesquiterpenes (1.70%), and other compounds (1.34%). The most abundant identified compounds were 6,9-guaiadiene (19.14%), *(E)*-caryophyllene (16.80%), bornyl acetate (14.74%), germacrene D (10.22%), *(E)*-β-farnesene (10.00%), and bicyclogermacrene (7.52%) (Table 1). Comparable results were obtained for the analysis performed on the polar column (Table 1). The percentage content of each compound reported in Table 1 is the mean of three analyses.

The chemical profiles of the EO from *S. leucantha* collected in Southern Ecuador (Table 1)**,** were compared with studies on samples from the plant collected in Asia and America. In these oils, sesquiterpenes and monoterpenes were reported as the major components. The EO from aerial parts including flowers of the plant collected in India, contained bornyl acetate (23.9%), *(E)*-caryophyllene (13.9%), germacrene D (13.8%), and bicyclogermacrene (8.7%) as major constituents [8], whereas the leaf EO was characterized by the presence of bornyl acetate (27.8%) and *(E)*-caryophyllene (10.7%) [28]. On the other hand, the EO from leaves of *S. leucantha* collected in Indonesia contained *(E)*-caryophyllene (7.87%) and bicyclogermacrene (5.13%) as the major components [9]. Bornyl acetate (11.4%), *(E)*-caryophyllene (6.5%), and 6,9-guaiadiene (4.9%) were the major EO components from aerial parts of *S. leucantha,* collected in United States [29]. A study of the EO from flowers collected in Colombia reported the presence of *(E)*-β-farnesene (10.7%), germacrene D (10.6%), *(E)*-caryophyllene (10%), and bornyl acetate (9.8%) [30]. Finally, a study of the EO from aerial parts (fresh leaves) of the plant collected in Venezuela, mentioned bornyl acetate (24.1%), *(E)*-caryophyllene (14.1%), bicyclogermacrene (8.9%), and germacrene D (6.6%) as the major constituents [5].

Comparing the compositions of the EOs from the different samples of *S. leucantha*, it appears that they are characterized by the presence of high amounts of sesquiterpenoids. However, the presence of a high amount (about 19%) of 6,9-guaiadiene is a special characteristic of the EO from aerial parts of the plant collected in Ecuador. In fact, significant amounts of this sesquiterpene have not been reported in the EOs of samples from India, Indonesia, Colombia and Venezuela, whereas the EO from *S. leucantha* aerial parts collected in USA contained less than 5% 6,9-guaiadiene. By contrast, significant amounts of (*E*)-caryophyllene and monoterpene bornyl acetate occur in almost all the EOs of *S. leucantha* examined so far.

In conclusion, the chemical profiles of the EOs isolated from *S. leucantha* growing in different countries are similar; however, they are not identical, and thus characterize each oil. On the other hand, one must remember that the different percentages of the major constituents of the different EOs could depend on several factors such as origin, age, plant part, climate, place of growth, soil conditions, temperature, extraction methods and storage [31].

The biological activities shown by the main constituents of *S. leucantha* EO justify the potential use of the plant as a medicinal remedy [32]. (*E*)-caryophyllene has exhibited anti-inflammatory, antidiabetic and hepatoprotective effects [33], antinociceptive activity [34] and relevant cytotoxicity against several types of cancer cells [35]. Germacrene D is involved in ecological interactions of plants with insects and other predators [36]; in addition, germacrene D has displayed anti-inflammatory properties [37]. *(E)*-β-Farnesene is currently used as a defense chemical against insects [38,39]; bicyclogermacrene showed cytotoxic activity [40] and a possible antiviral activity against SARS-CoV-2 [41]; finally, bornyl acetate exhibited antioxidant [42], anti-inflammatory [43,44] and repellent effects against storage insects [45].

### 2.3. Enantiomeric Analysis of the EO

The enantiomeric composition of an EO is an important characteristic of the oil and an essential marker to determine the authenticity of a plant species [46]. Therefore, we submitted for the first time the EO from *S. leucantha* to an enantioselective analysis. Using the chiral column at our disposal we detected four pairs of enantiomers whose peaks were well separated at the base and could be quantified with accuracy. The linear retention indices, enantiomeric distribution and enantiomeric excess (*ee*%) of each pair are shown in Table 2. The order of elution of the enantiomers was determined by injection of enantiomerically pure standards.

(−)-Germacrene D and (+)-α-pinene exhibited a high enantiomeric excess, whereas (−)-aromadendrene was enantioenriched only moderately and the enantiomeric excess of (−)-sabinene was low.

The majority of the EO components detected by the GC-FID analysis (Table 1), including the most abundant ones, overlapped on our chiral column or appeared as a single peak. Of course, this finding did not necessarily indicate that they were enantiomerically pure, because peak coalescence might depend on the inadequate resolving power of the chiral column used. However, although the enantioselective analysis of the EO from *S. leucantha* cannot be considered definitive, these data are, however, significant and add further evidence that secondary metabolites often exist in plants as mixtures of stereoisomers.

### 2.4. Cholinesterase (AChE and BuChE) Inhibitory Activity of the EO

The enzymes AChE as well as BuChE are considered to be primary ChE regulators [47]; moreover, cholinesterase (ChE) inhibition represents the most efficacious treatment approach for Alzheimer’s disease (AD) to date. In a normal brain, AChE represents 80% of the activity, while BuChE represents the remaining 20% [48]. In the brains of patients with Alzheimer’s disease (AD) brain, BuChE activity rises, while AChE activity remains unchanged or declines. It has been demonstrated that the selective inhibition of BuChE not only increases the acetylcholine level significantly but also improves memory in elderly rats [49]. Therefore, ChE inhibitors, as well as selective BuChE inhibitors derived from nature, are actively searched for in different laboratories worldwide. In this context, the neuroprotective and anti-ageing potentials of essential oils (EOs) are considered to be highly effective and are therefore widely evaluated [50].

**Figure 1:**
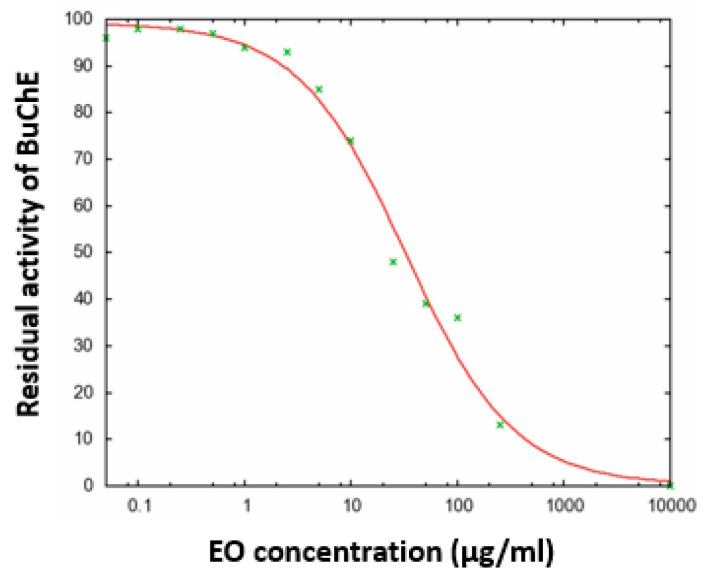
Inhibition activity curve of *S. leucantha* EO against BuChE.

In our continuing search for selective ChE inhibitors from natural sources, we have tested the *S. leucantha* EO in an in vitro assay (see Experimental). The oil showed high activity against BuChE, with a value of IC_50_ = 32.60 ± 5.60 µg/mL (Figure 1), while it was inactive, IC_50_ > 250 µg/mL, against the enzyme AChE.

In comparison with these results, other *Salvia* species, i.e., *S. pichinchensis* [17], *S. fruticosa*, and *S. officinalis* [51], showed significantly lower BuChE inhibitory activity, with values of IC_50_ = 50.70 µg/mL, 150 µg/mL and 140 µg/mL, respectively.

## 3. Materials and Methods

### 3.1. Collection of Plant Material

Aerial parts of *Salvia leucantha* Cav. in full bloom were collected in June 2019 in province of Azuay, Cuenca, Ecuador (2°55′05.8″ S 7°900′00.9″ O). The plant was identified by the UTPL botanist José Miguel Andrade and a voucher has been deposited in the UTPL herbarium with the code HUTPL14256.

### 3.2. Essential Oil Isolation

The EO of *S. leucantha* was obtained from the aerial part of the plant (leaves and flowers) by steam distillation for 4 h, using a Clevenger-type equipment. The EO was separated from the aqueous layer, dried over anhydrous Na_2_SO_4_, filtered, and stored in an amber vial at 4 °C until use. The procedure was performed in triplicate.

### 3.3. Physical Properties

The relative density of the EO was determined according to the international standard AFNOR NF T75-111 (ISO 279:1998) guidelines. The refractive index was determined in an ABBE refractometer, according to the international standard AFNOR NF 75-112 (ISO 280:1998) procedure. Each analysis was performed in triplicate.

### 3.4. Chemical Composition of the Essential Oil

#### 3.4.1. Gas Chromatography Coupled to Mass Spectrometry (GC-MS)

The EO was qualitatively analysed by GC-MS, using an Agilent Technologies Chromatograph 6890N, coupled to an Agilent mass spectrometer, 5973 Inert series, which operated in electron impact mode at a 70 eV and was controlled by an MSD-ChemStation software.

Two types of chromatographic columns were used for the analysis: a non-polar column DB-5ms (5% phenyl-methylpolysiloxane; 30 m × 0.25 mm × 0.25 µm) and a HP-INNOWax polar column (polyethylene glycol; 30 m × 0.25 mm × 0.25 µm); helium was used as the carrier gas in both analyses (at a constant flow of 1.00 mL/min). The injection system operated in split mode (40:1) at 220 °C. The GC oven temperature was programmed at 60 °C (5 min), then it was increased to 250 °C (10 min) at a rate of 3 °C/min. We injected 1 μL of a solution of the EO in CH_2_Cl_2_ (1:100 *v*/*v*) in each analysis.

The volatile components of the EO were identified by comparing the linear retention indices (LRI) and the mass spectra with the data reported in the literature. Each LRI was calculated according to Van Den Dool and Kratz [27], from a homologous series of *n*-alcanes C_9_–C_25_ (C_9_, BHD purity 99% and C_10_–C_25_, Fluka purity 99%), which were injected on each of the two columns after the EO, under identical conditions [17].

#### 3.4.2. Gas Chromatography Coupled to the Flame Ionization Detector (GC-FID)

Quantitative analysis of the EO was carried out by GC-FID on an Agilent Technologies 6890N series gas-chromatograph coupled to a flame ionization detector (FID), using the DB-5ms and HP-INNOWax columns cited before. The analytical gas-chromatographic conditions were similar to those used for the GC-MS analysis. The relative amount (expressed as a percentage) of each compound identified in the EO was calculated by comparing the area of the corresponding peak in the GC-FID chromatogram with the total area of identified peaks (Table 1). No correction factor was applied. Three injections of the EO were performed to determine each average percentage and the standard deviation [17].

### 3.5. Enantiomeric Analysis

The enantioselective analysis of the EO was performed on the GC-MS instrument cited before, using the chiral capillary column MEGA-DEX-DET-Beta (Diethyl tertbutylsilyl-β-cyclodextrin; 25 m × 0.25 mm × 0.25 μm). The analytical conditions were the same as those used for the qualitative analysis of the oil, except the oven temperature, which was kept at 60 °C (5 min), then increased to 220 °C (5 min) at a rate of 2 °C/min. The enantiomeric distribution and enantiomeric excess of each enantiomeric pair were determined by comparison with authentic reference compounds.

### 3.6. Inhibition of Cholinesterase (AChE and BuChE)

The inhibitory activity of cholinesterases (ChEs) by the EO was determined by a colorimetric process based on the methodology established by Ellman et al. [52], against acetylcholinesterase (AChE, from *Electrophorus electricus*, Sigma Aldrich, C3389, St. Louis, MO, USA) and butyrylcholinesterase (BuChE, from an equine serum, Sigma Aldrich, SRE020, Missouri, USA). The two enzymes catalyze the hydrolysis of choline esters such as acetylthiocholine (ACh) [53]. A phosphate-buffered saline solution (200 μL, pH 7.4), DTNB or Ellman reagent (1.5 mM) and an EO sample dissolved in DMSO (1% *v*/*v*) were used for the test. The enzymes AChE and BuChE were separately dissolved in the phosphate-buffered saline solution, using 25 mU/mL for each test. After 10 min of pre-incubation, acetylthiocholine iodide (1.5 mM) was added to start the reaction. After 30 min of incubation at 30 °C, the reaction kinetics were read in 96-well microtiter plates by a Varioskan Flash (Thermo Fisher Scientific, Waltham, MA, USA) detection system. Enzymatic activities were calculated for increasing concentrations, from 0.05 to 250 µg/mL (see Figure 1) of the EO dissolved in DMSO. For the assay, false-positive results (>100 µg/mL), due to the possible presence of amines or aldehydes, were excluded [54]. All measurements were performed in triplicate. The IC_50_ inhibition values were calculated using a nonlinear regression model (GNUPLOT package, www.ic50.tk (accessed on 1 March 2021), www.gnuplot.info (accessed on 1 March 2021)). Donepezil was used as a reference inhibitor [55] for both enzymes, AChE (IC_50_ = 0.04 µg/mL) and BuChE (IC_50_ =3.6 µg/mL).

## 4. Conclusions

The EO steam-distilled from the aerial parts of *S. leucantha,* collected in Southern Ecuador, contained a high amount of sesquiterpene hydrocarbons. The most abundant components were 6.9-guaiadiene, *(E)*-caryophyllene, bornyl acetate, germacrene D, *(E)*-β-farnesene, and bicyclogermacrene. The enantioselective analysis of the oil, performed for the first time, indicated the presence of four non-racemic pairs of enantiomers. Moreover, the EO of *S. leucantha* exhibited an interesting selective inhibitory activity against the enzyme BuChE. The value of IC_50_ = 32.60 µg/mL is possibly the highest for EOs from *Salvia* species [51]. The oil can therefore be considered as a source of bioactive phytochemicals and phytotherapeutics for humans due to its potential anti-Alzheimer activity.

## Figures and Tables

**Table 1:** Chemical composition of *Salvia leucantha* Cav. EO.

Component	DB-5ms				HP-INNOWax			
	LRI ^a^	LRI ^b^ [16]	% ^c^	S.D ^d^	LRI ^a^	LRI ^b^	% ^c^	S.D ^d^
α-Pinene	942	932	3.31	0.68	1055	1066 [17]	3.70	0.31
Camphene	956	946	3.03	0.55	1079	1084 [17]	3.33	0.27
Sabinene	975	969	0.48	0.07	1120	1117 [18]	0.51	0.06
β-Pinene	979	974	2.01	0.50	1108	1104 [18]	2.15	0.17
Myrcene	990	988	0.17	0.02	-	-	-	-
Limonene	1028	1024	0.35	0.07	1199	1199 [17]	0.29	0.03
γ-Terpinene	1056	1054	0.13	0.02	-	-	-	-
α-Phellandrene	-	-	-	-	1162	1160 [19]	0.15	0.01
3-Methyl-3-butenyl, 3-methyl- butanoate	1112	1112	0.12	0.00	-	-	-	-
Borneol	1171	1165	0.14	0.04	-	-	-	-
Terpinene-4-ol	1178	1174	0.12	0.04	-	-	-	-
Bornyl acetate	1286	1284	14.74	0.31	1577	1580 [20]	13.26	0.22
Humulene	-	-	-	-	1658	1660 [20]	0.75	0.01
δ-Elemene	1333	1335	0.54	0.01	1466	1460 [20]	1.30	0.18
α-Copaene	1372	1374	0.36	0.01	1483	1493 [18]	0.35	0.00
β-Bourbonene ^e^	1379	1387	0.35	0.00	1509	1519 [21]	0.36	0.01
β-Elemene	1386	1389	0.42	0.01	-	-	-	-
*(E)*-Caryophyllene	1418	1417	16.80	0.16	1588	1590 [20]	17.56	0.20
β-Copaene	1425	1430	0.14	0.01	-	-	-	-
Aromadendrene	1433	1439	0.35	0.00	1613	1613 [22]	1.74	0.01
6,9-Guaiadiene	1442	1442	19.14	0.29	1600	1617 [23]	17.8	0.17
Allo-aromadendrene	1447	1458	1.88	0.04	1633	1633 [17]	0.64	0.01
*(E)*-β-Farnesene	1455	1454	10.00	0.47	1670	1665 [24]	9.11	0.16
Germacrene D	1481	1480	10.22	0.11	1699	1700 [20]	12.5	0.20
*trans*-Cadina-1(6).4-diene	1482	1475	0.53	0.00	-	-	-	-
β-Selinene	1485	1489	0.29	0.16	1706	1708 [24]	0.38	0.04
Bicyclogermacrene	1494	1500	7.52	0.07	1724	1723 [17]	6.23	0.04
*n*-Pentadecane	1500	1500	0.16	0.01	1500	1500 [25]	0.26	0.01
γ-Cadinene	1509	1513	1.07	0.02	1750	1750 [17]	0.47	0.01
δ-Amorphene	1511	1511	1.31	0.05	-	-	-	-
Furopelargone A ^e^	1527	1538	0.39	0.02	-	-	-	-
Germacrene B	1553	1559	0.41	0.03	1815	1811 [26]	0.24	0.02
Spatulenol	1573	1577	0.55	0.00	2118	2118 [17]	1.90	0.07
Cubeban-11-ol ^e^	1577	1595	0.62	0.01	-	-	-	-
Cadinol	1638	1638	0.14	0.00	-	-	-	-
Mint sulfide ^e^	1730	1740	0.29	0.06	-	-	-	-
Sclarene	1977	1974	1.05	0.15	-	-	-	-
Caryophyllene oxide	-	-	-	-	1967	1967 [17]	0.87	0.01
α-Cadinol	-	-	-	-	2229	2220 [21]	0.22	0.01
*trans*-α-Bergamotol	-	-	-	-	2249	2247 [23]	1.98	0.19
Monoterpene hydrocarbons (%)			9.48				10.13	
Oxygenated monoterpenes (%)			15.12				13.26	
Sesquiterpene hydrocarbons (%)			71.49				69.69	
Oxygenated sesquiterpenes (%)			1.70				4.97	
Others (%)			1.34				-	
Total identified (%)			99.13				98.05	

^a^ Linear retention index (LRI), calculated according to Van den Dool and Kratz [27]; ^b^ reference linear retention index; ^c^ content (%) as a mean of three determinations; ^d^ standard deviation; ^e^ identified tentatively.

**Table 2:** Enantiomeric analysis of *Salvia leucantha* Cav. EO.

Enantiomer	LRI ^a^	Enantiomeric Distribution (%)	*ee%* ^b^
(+)-α-Pinene	926	4.32	
			91.36
(−)-α-Pinene	932	95.68	
(+)-Sabinene	973	29.98	
			40.04
(−)-Sabinene	975	70.02	
(+)-Aromadendrene	1426	19.15	
			61.70
(−)-Aromadendrene	1440	80.85	
(+)-Germacrene D	1475	1.65	
			96.70
(−)-Germacrene D	1489	98.35	

^a^ Linear retention index calculated on the chiral capillary column diethyl tertbuthysilyl-β-cyclodextrin; ^b^ enantiomeric excess.
